# Association of red cell distribution width/albumin ratio and in hospital mortality in patients with atrial fibrillation base on medical information mart for intensive care IV database

**DOI:** 10.1186/s12872-024-03839-6

**Published:** 2024-03-21

**Authors:** Li-ya Pan, Jing Song

**Affiliations:** Department of Cardiology, The Second Affiliated Hospital, Yuying Children’s Hospital of Wenzhou Medical University, Wenzhou, 325000 China

**Keywords:** Atrial fibrillation, Mortality, Inflammation, Red cell distribution width, Albumin

## Abstract

**Background:**

Atrial fibrillation (AF) is a common cardiac arrhythmia. The ratio of red cell distribution width (RDW) to albumin has been recognized as a reliable prognostic marker for poor outcomes in a variety of diseases. However, the evidence regarding the association between RDW to albumin ratio (RAR) and in hospital mortality in patients with AF admitted to the Intensive Care Unit (ICU) currently was unclear. The purpose of this study was to explore the association between RAR and in hospital mortality in patients with AF in the ICU.

**Methods:**

This retrospective cohort study used data from the Medical Information Mart for Intensive Care IV (MIMIC-IV) database for the identification of patients with atrial fibrillation (AF). The primary endpoint investigated was in-hospital mortality. Multivariable-adjusted Cox regression analysis and forest plots were utilized to evaluate the correlation between the RAR and in-hospital mortality among patients with AF admitted to ICU. Additionally, receiver operating characteristic (ROC) curves were conducted to assess and compare the predictive efficacy of RDW and the RAR.

**Results:**

Our study included 4,584 patients with AF with a mean age of 75.1 ± 12.3 years, 57% of whom were male. The in-hospital mortality was 20.3%. The relationship between RAR and in-hospital mortality was linear. The Cox proportional hazard model, adjusted for potential confounders, found a high RAR independently associated with in hospital mortality. For each increase of 1 unit in RAR, there is a 12% rise in the in-hospital mortality rate (95% CI 1.06–1.19). The ROC curves revealed that the discriminatory ability of the RAR was better than that of RDW. The area under the ROC curves (AUCs) for RAR and RDW were 0.651 (95%CI: 0.631–0.671) and 0.599 (95% CI: 0.579–0.620).

**Conclusions:**

RAR is independently correlated with in hospital mortality and in AF. High level of RAR is associated with increased in-hospital mortality rates.

**Supplementary Information:**

The online version contains supplementary material available at 10.1186/s12872-024-03839-6.

## Introduction

Atrial fibrillation (AF) is the most prevalent cardiac arrhythmia, characterized by irregular and rapid atrial contractions [1, 2]. Its prevalence increases with age, and it is associated with various cardiovascular and cerebrovascular complications [3, 4]. AF significantly heightens the risk of stroke, heart failure, and mortality [4–7]. Although many risk factors for atrial fibrillation have already been identified, including age, hypertension, diabetes, obesity, left atrial volume and smoking [8–10], the potential modifiable risk factors still need to be investigated.

RDW is a relatively easily obtainable indicator, representing the heterogeneity in the size of circulating red blood cells [11]. It is commonly used for the differentiation of anemia [11]. Research has revealed associations between RDW and various diseases, including diabetes, pulmonary embolism, chronic obstructive pulmonary disease, heart failure, and cerebrovascular diseases [12–14]. Current studies indicate that an elevated RDW is linked to adverse cardiovascular outcomes, serving as a marker of inflammation and oxidative stress [14–16].

Albumin, the most abundant circulating protein in the blood, plays a crucial role in binding and transporting various drugs and substances. It contributes to maintaining blood osmolality and influencing the physiological functions of the circulatory system [17]. Extensive evidence indicates that albumin serves as a robust predictor of cardiovascular risk across diverse patient populations [18].

RDW/ALB has emerged as a composite marker, integrating inflammatory status (RDW) and nutritional status (albumin) .To date, there have been few studies on the prognostic value of the RDW/ALB ratio in AF [19]. Although RAR is a reliable indicator of mortality based on systemic inflammation in many diseases [20–22], there is insufficient evidence that RAR has predictive value for prognosis in AF patients. In this study, we sought to explore the association of RDW/ALB ratio on hospital mortality in a relatively large cohort of patients with atrial fibrillation.

## Materials and methods

### Data source

This retrospective study was based on the Medical Information Mart for Intensive Care IV database (MIMIC-IV, Version 2.2). The database comprises data from over 70,000 patients admitted to Beth Israel Deaconess Medical Center’s ICUs in Boston, MA, from 2008 to 2019.The database has received approval from the Institutional Review Boards of the Massachusetts Institute of Technology (Cambridge, MA, USA) and Beth Israel Deaconess Medical Center (Boston, MA, USA) [23]. To safeguard the privacy of patients included in the study, all personal information has been systematically removed. Individuals who have successfully completed the Collaborative Institutional Training Initiative exam are granted access to this database. Considering the retrospective nature of this study and the extraction of patient data from a public database, the requirement for informed consent has been waived.

### Study subjects

**Fig. 1:**
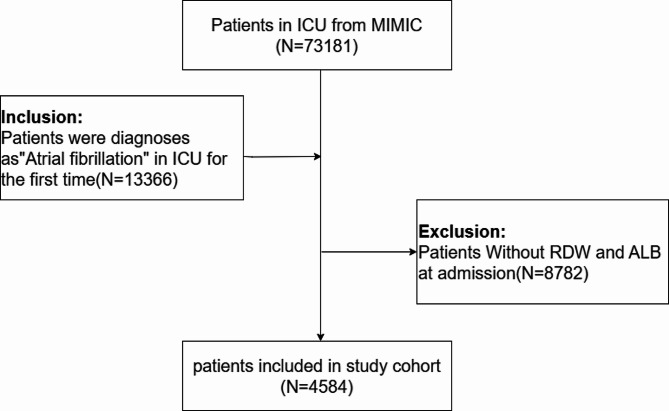
Flow chart of the study population Abbreviations: ICU, intensive care unit; MIMIC, Medical Information Mart for Intensive Care IV; RDW, red cell distribution width; ALB, albumin

Patients for this study were identified within the MIMIC-IV database from 2008 to 2019. The study population consisted of individuals diagnosed with atrial fibrillation (AF) and subsequently admitted to the intensive care unit (ICU). The diagnosis of AF was established through the International Classification of Diseases (ICD) codes (Table S1 in Supplementary Appendix). Inclusion criteria were defined as follows: patients with atrial fibrillation admitted to the ICU for the first time (*N* = 13,366). 8,782 patients lacking Red Cell Distribution Width (RDW) and Albumin (ALB) measurements were excluded. Ultimately, a cohort of 4,584 patients with complete data on RDW and ALB, diagnosed with atrial fibrillation, were included in this study (Fig. 1).

### Demographical and laboratory variables

The MIMIC-IV database was queried for patient information using structured query language (SQL). Data extraction included information such as population statistics (age, gender, height and weight), vital signs (respiratory rate, heart rate, systolic and diastolic blood pressure), comorbidities (hypertension, diabetes, myocardial infarction, heart failure, cerebrovascular disease, and chronic pulmonary disease), and laboratory parameters (minimum hemoglobin and platelet counts, maximum white blood cell count, creatinine, blood urea nitrogen, glucose, lactate, ALT, AST, INR, albumin, RDW). In instances where an indicator had multiple records, the mean measured value was used. The Body Mass Index (BMI) was computed by dividing body weight (kg) by the square of height (m).

### RAR assessment and outcomes

RAR was calculated using the following formula: [RDW (%)/serum albumin (g/dL)]. The study’s outcome focused on in-hospital mortality after admission to the ICU.

### Statistical analysis

Continuous variable data were described as mean ± standard deviation (SD) or median and interquartile range (IQR), while categorical variable data were described as frequencies or percentages. Baseline characteristics underwent comparison using the Mann–Whitney test for continuous variables and the chi-square test for categorical variables. Multivariate logistic regression analysis was executed to evaluate the association between RAR and in-hospital mortality in individuals with AF. Results were conveyed as odds ratios (OR) with 95% confidence intervals (CI). RAR values were categorized into quartiles, with the first quartile serving as the reference group. Four models were applied in the regression analysis, adjusting for diverse factors: Model 1 adjusted for age, gender, and BMI; Model 2 for age, gender, BMI, sbp, dbp, heart rate, and respiratory rate; Model 3 for Model 2 plus hypertension, diabetes, myocardial infarction, congestive heart failure, cerebrovascular disease, and chronic pulmonary disease; and Model 4 for Model 3 plus hemoglobin and platelet counts, white blood cell count, creatinine, blood urea nitrogen, glucose, lactate, alanine aminotransferase(ALT), aspartate aminotransferase(AST), international normalized ratio(INR). The predictive performance of RDW and RDW/ALB ratio was assessed through pairwise Receiver Operating Characteristic (ROC) curve analyses. Additionally, subgroup analysis was conducted to assess whether there were differences in the impact of RAR on in-hospital mortality rates among different subgroups of patients with AF.

All analyses were per formed using R 4.2.2 (http://www.R-project.org, R Foundation) and Free Statistics version 1.9, *P* < 0.05 was considered statistically significant.

## Results

### Baseline characteristics of study subjects

After screenings, the presented study included 4,584 MIMIC-IV patients with AF. The baseline characteristics were classified according to RDW/ALB quartiles (Table 1). In MIMIC-IV, there were 1,146 patients in quartile 1(Q1), 1,146 patients in quartile 2(Q2), 1,142 patients in quartile 3(Q3), and 1,150 patients in quartile 4(Q4). There were 1,969 females and 2,615 males in these patients. Patients in the high quartile group had lower systolic and diastolic blood pressure, faster heart rate, lower hemoglobin and albumin levels, higher leukocytes, blood creatinine, urea nitrogen, lactate, ALT, AST, INR, and RDW, and higher in-hospital mortality rates.

**Table 1:** Baseline characteristics of the study participants

Characteristics	Total (*n* = 4584)	Q1(< 3.93) (*n* = 1146)	Q2 (3.93–4.73) (*n* = 1146)	Q3 (4.73–5.83) (*n* = 1142)	Q4 (> 5.83) (*n* = 1150)	P-value
Age(years)	75.1 ± 12.3	74.9 ± 12.2	76.4 ± 12.2	75.5 ± 12.0	73.8 ± 12.5	< 0.001
gender, n (%)						0.469
Female	1969 (43.0)	479 (41.8)	511 (44.6)	497 (43.5)	482 (41.9)	
Male	2615 (57.0)	667 (58.2)	635 (55.4)	645 (56.5)	668 (58.1)	
BMI (kg/m^2)^	29.3 ± 8.0	29.2 ± 6.8	29.0 ± 7.9	29.2 ± 8.3	29.7 ± 8.6	0.493
SBP (mmHg)	116.8 ± 17.2	124.6 ± 17.9	118.6 ± 16.8	114.1 ± 16.0	110.0 ± 14.3	< 0.001
DBP (mmHg)	62.9 ± 11.4	67.5 ± 12.4	63.4 ± 11.1	61.2 ± 10.5	59.4 ± 9.8	< 0.001
Heart rate, beats/min	86.8 ± 17.9	82.2 ± 17.1	85.9 ± 17.4	88.3 ± 17.9	90.9 ± 18.1	< 0.001
Respiratory rate, beats/min	20.1 ± 3.8	19.5 ± 3.3	20.1 ± 3.6	20.4 ± 4.0	20.5 ± 4.3	< 0.001
hypertension, n (%)						< 0.001
No	4049 (88.7)	902 (78.8)	1010 (88.6)	1038 (91.6)	1099 (96)	
Yes	514 (11.3)	243 (21.2)	130 (11.4)	95 (8.4)	46 (4)	
Diabetes, n (%)						0.025
No	3404 (74.3)	871 (76)	872 (76.1)	814 (71.3)	847 (73.7)	
Yes	1180 (25.7)	275 (24)	274 (23.9)	328 (28.7)	303 (26.3)	
Myocardial infarct, n (%)						0.73
No	3525 (76.9)	888 (77.5)	877 (76.5)	867 (75.9)	893 (77.7)	
Yes	1059 (23.1)	258 (22.5)	269 (23.5)	275 (24.1)	257 (22.3)	
Congestive heart failure, n (%)						< 0.001
No	2354 (51.4)	699 (61)	527 (46)	549 (48.1)	579 (50.3)	
Yes	2230 (48.6)	447 (39)	619 (54)	593 (51.9)	571 (49.7)	
Cerebrovascular disease, n (%)						< 0.001
No	3629 (79.2)	767 (66.9)	895 (78.1)	974 (85.3)	993 (86.3)	
Yes	955 (20.8)	379 (33.1)	251 (21.9)	168 (14.7)	157 (13.7)	
Chronic pulmonary disease, n (%)						< 0.001
No	3251 (70.9)	887 (77.4)	762 (66.5)	778 (68.1)	824 (71.7)	
Yes	1333 (29.1)	259 (22.6)	384 (33.5)	364 (31.9)	326 (28.3)	
hemoglobin/dl	10.1 ± 2.3	11.6 ± 2.1	10.4 ± 2.1	9.6 ± 2.1	8.8 ± 1.9	< 0.001
WBC,10^9^/L	15.1 ± 13.8	13.1 ± 9.0	14.2 ± 11.5	15.6 ± 16.0	17.4 ± 16.8	< 0.001
platelets, 10^9^/L	183.8 ± 99.0	187.4 ± 76.1	188.1 ± 93.9	183.2 ± 99.0	176.5 ± 121.2	0.018
creatinine, mg/dl	1.3 (0.9, 2.1)	1.1 (0.8, 1.5)	1.3 (0.9, 1.9)	1.5 (1.0, 2.5)	1.6 (1.0, 2.7)	< 0.001
BUN, mg/dl	29.0 (19.0, 48.0)	22.0 (16.0, 31.0)	27.0 (19.0, 46.0)	35.0 (22.0, 55.0)	36.0 (23.0, 58.0)	< 0.001
glucose, mg/dl	150.0 (119.0, 203.5)	146.0 (118.0, 191.8)	149.0 (119.0, 199.0)	153.0 (122.0, 208.8)	152.0 (119.0, 211.0)	0.049
lactate, mmol/L	2.3 (1.5, 4.2)	2.2 (1.5, 3.4)	2.1 (1.4, 3.5)	2.3 (1.4, 4.4)	2.8 (1.6, 5.8)	< 0.001
ALT, U/L	26.0 (16.0, 61.0)	23.0 (16.0, 41.0)	25.0 (16.0, 59.0)	29.0 (17.0, 75.0)	32.0 (16.0, 81.5)	< 0.001
AST, U/L	39.0 (24.0, 90.0)	32.0 (23.0, 56.0)	37.0 (24.0, 82.0)	45.0 (25.0, 110.0)	50.0 (26.0, 133.0)	< 0.001
INR	1.5 (1.2, 2.1)	1.3 (1.1, 1.7)	1.4 (1.2, 2.2)	1.5 (1.3, 2.2)	1.6 (1.3, 2.3)	< 0.001
Albumin, g/dl	3.2 ± 0.7	3.9 ± 0.3	3.4 ± 0.3	3.0 ± 0.4	2.5 ± 0.5	< 0.001
RDW, %	15.5 ± 2.3	13.7 ± 0.9	14.8 ± 1.4	15.9 ± 1.9	17.5 ± 2.8	< 0.001
RAR [%/(g/dl)]	5.1 ± 1.6	3.5 ± 0.3	4.3 ± 0.2	5.2 ± 0.3	7.3 ± 1.5	< 0.001
hospital mortality, n (%)						< 0.001
No	3652 (79.7)	1017 (88.7)	959 (83.7)	903 (79.1)	773 (67.2)	
Yes	932 (20.3)	129 (11.3)	187 (16.3)	239 (20.9)	377 (32.8)	

Data were presented as n (%), mean (SD) and median (IQR).

SD, standard deviation; IQR, interquartile range; BMI, body mass index; SBP, systolic blood pressure; DBP, diastolic blood pressure; WBC, white blood cell; BUN, blood urea nitrogen; ALT, alanine aminotransferase; AST, aspartate aminotransferase; INR, international normalized ratio; RDW, red cell distribution width; RAR, RDW to albumin ratio

### Associations between RAR and mortality

**Fig. 2:**
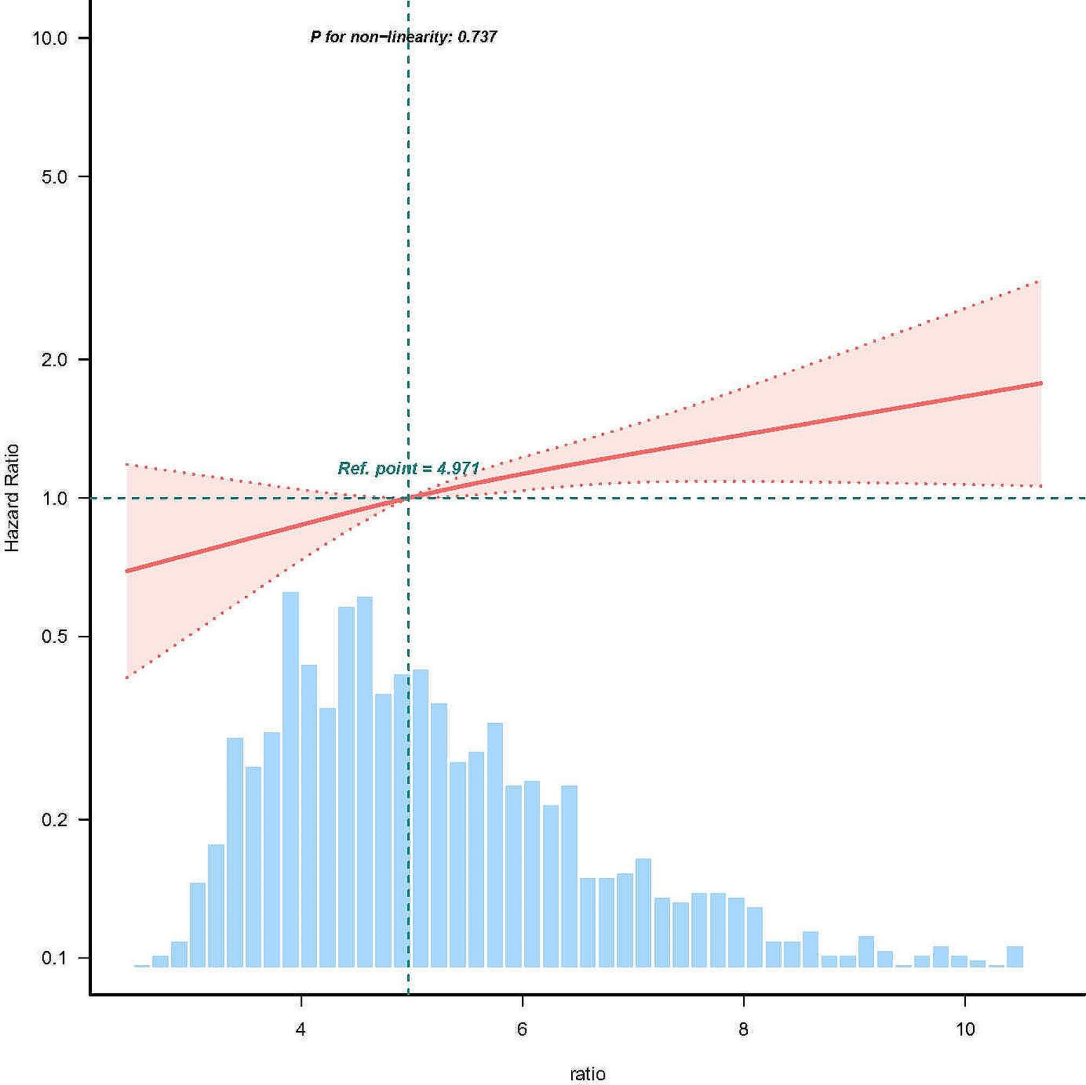
linear dose-response relationship between RAR and in hospital mortality of patients with atrial fibrillation. Adjustment factors included age, gender, BMI, sbp, dbp, Heart rate, respiratory rate, hypertension, diabetes, myocardial infarct, congestive heart failure, cerebrovascular disease, chronic pulmonary disease, hemoglobin, WBC, platelets, creatinine, BUN, glucose, lactate, ALT, AST, INR.

There is a significant positive linear association between RAR and in hospital mortality in patients with AF (p for non-linearity > 0.05, Fig. 2). The results of the multivariate Cox regression analysis indicate a significant association between RAR and in-hospital mortality in patients with atrial fibrillation (AF). The odds ratios (OR) of RAR were significant in all models when RAR is considered as a continuous variable (*p* < 0.001). For each increase of 1 unit in RAR, there is a 12% rise in the in-hospital mortality rate (95% CI 1.06–1.19). Furthermore, when RAR is categorized into quartiles and adjusting for various factors including age, gender, body mass index (BMI), heart rate, respiratory rate, systolic and diastolic blood pressure, hypertension, diabetes, congestive heart failure, myocardial infarction, cerebrovascular disease, chronic pulmonary disease, and laboratory indicators, a notable trend is observed. As RAR quartiles increase, the in-hospital mortality rate exhibits a corresponding increase. (p for trend < 0.05) (Table 2).

**Table 2:** Unadjusted and multivariate cox regression analyses for in hospital mortality

Outcome	Non-adjusted Model		Model I		Model II		Model III		Model IV	
	OR(95%CI)	P-value	OR(95%CI)	P-value	OR(95%CI)	P-value	OR(95%CI)	P-value	OR(95%CI)	P-value
RAR [%/(g/L)]	1.16 (1.13,1.20)	< 0.001	1.15 (1.1,1.19)	< 0.001	1.12 (1.07,1.17)	< 0.001	1.12 (1.07,1.17)	< 0.001	1.12 (1.06,1.19)	< 0.001
RAR quartiles										
Q1	1(Ref)		1(Ref)		1(Ref)		1(Ref)		1(Ref)	
Q2	1.23 (0.98,1.54)	0.074	1.28 (0.96,1.72)	0.091	1.2 (0.89,1.60)	0.234	1.25 (0.93,1.68)	0.136	1.57 (1.06,2.32)	0.024
Q3	1.46 (1.18,1.81)	0.001	1.46 (1.1,1.92)	0.008	1.26 (0.95,1.68)	0.112	1.31 (0.99,1.75)	0.062	1.66 (1.13,2.43)	0.009
Q4	1.94 (1.59,2.38)	< 0.001	1.9 (1.46,2.47)	< 0.001	1.56 (1.18,2.05)	0.002	1.6 (1.21,2.11)	0.001	1.79 (1.23,2.61)	0.002
P for trend		< 0.001		< 0.001		0.001		0.001		0.007

Crude model: no other covariates were adjusted

Model I: Adjust for age, gender and BMI

Model II: Adjust for age, gender, BMI. sbp, dbp, Heart rate, respiratory rate

Model III: Adjust for age, gender, BMI. sbp, dbp, Heart rate, respiratory rate, hypertension, diabetes, myocardial infarct, congestive heart failure, cerebrovascular disease, chronic pulmonary disease

Model IV: Adjust for Model III plus hemoglobin, WBC, platelets, creatinine, BUN, glucose, lactate, ALT, AST, INR

OR, odd ratio; CI, confidence interval; Ref, reference; BMI, body mx index; WBC, white blood cell; SBP, systolic blood pressure; DBP, diastolic blood pressure; BUN, blood urea nitrogen; ALT, alanine aminotransferase; AST, aspartate aminotransferase; INR, international normalized ratio; RDW, red cell distribution width; RAR, RDW to albumin ratio

### Subgroup Analysis

**Fig. 3:**
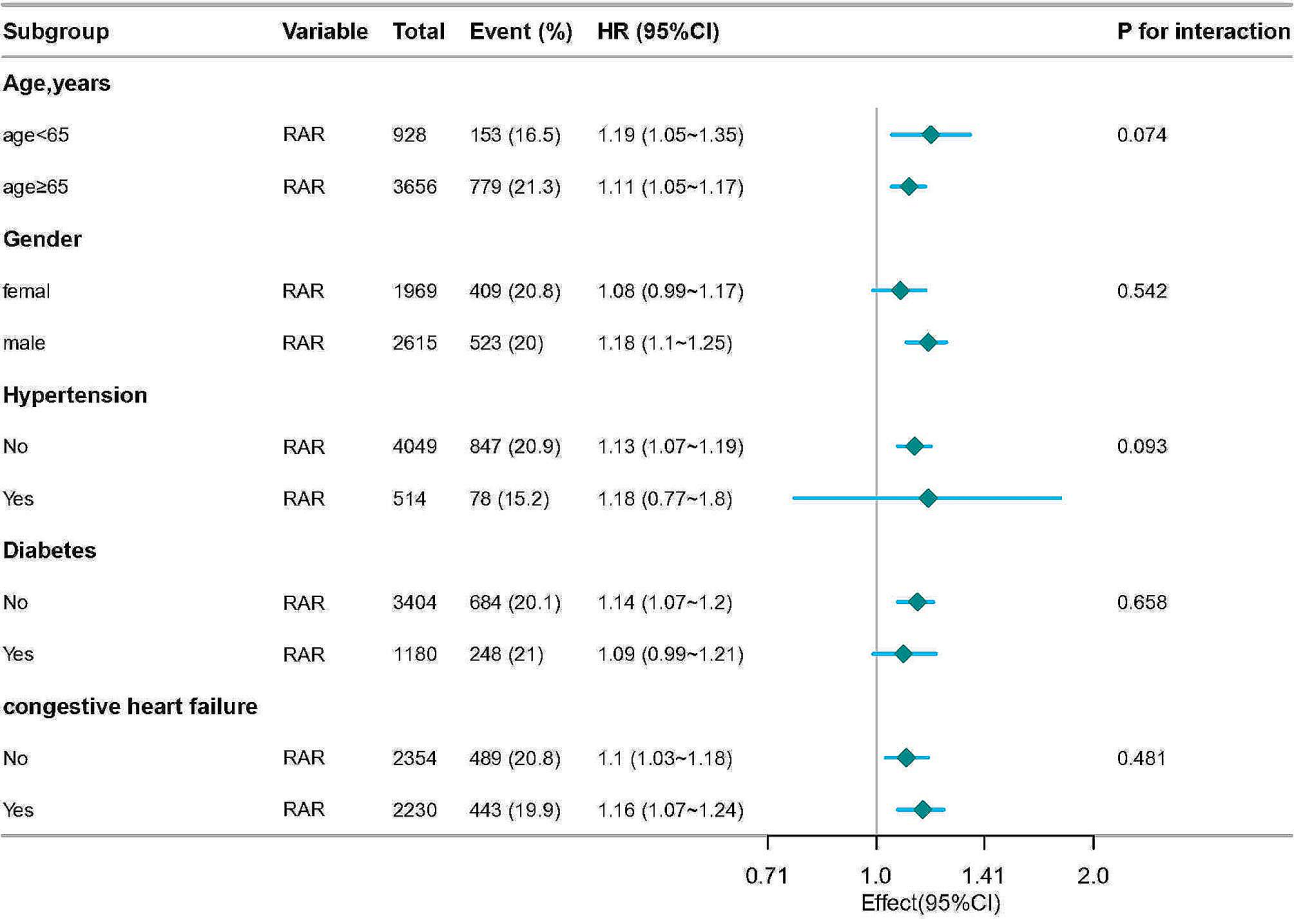
Subgroup analysis of relationships between RAR and in hospital mortality among AF patients ORs were adjusted for age, gender, BMI, sbp, dbp, Heart rate, respiratory rate, hypertension, diabetes, myocardial infarct, congestive heart failure, cerebrovascular disease, chronic pulmonary disease, hemoglobin, WBC, platelets, creatinine, BUN, glucose, lactate, ALT, AST, INR

We analyzed several subgroups, including age, gender, hypertension, diabetes, and heart failure. The impact of RAR on in-hospital mortality rates was found to be consistent across these subgroups. Additionally, no interaction effects were observed among the different subgroups (Fig. 3).

### Predictive performance of RAR

**Fig. 4:**
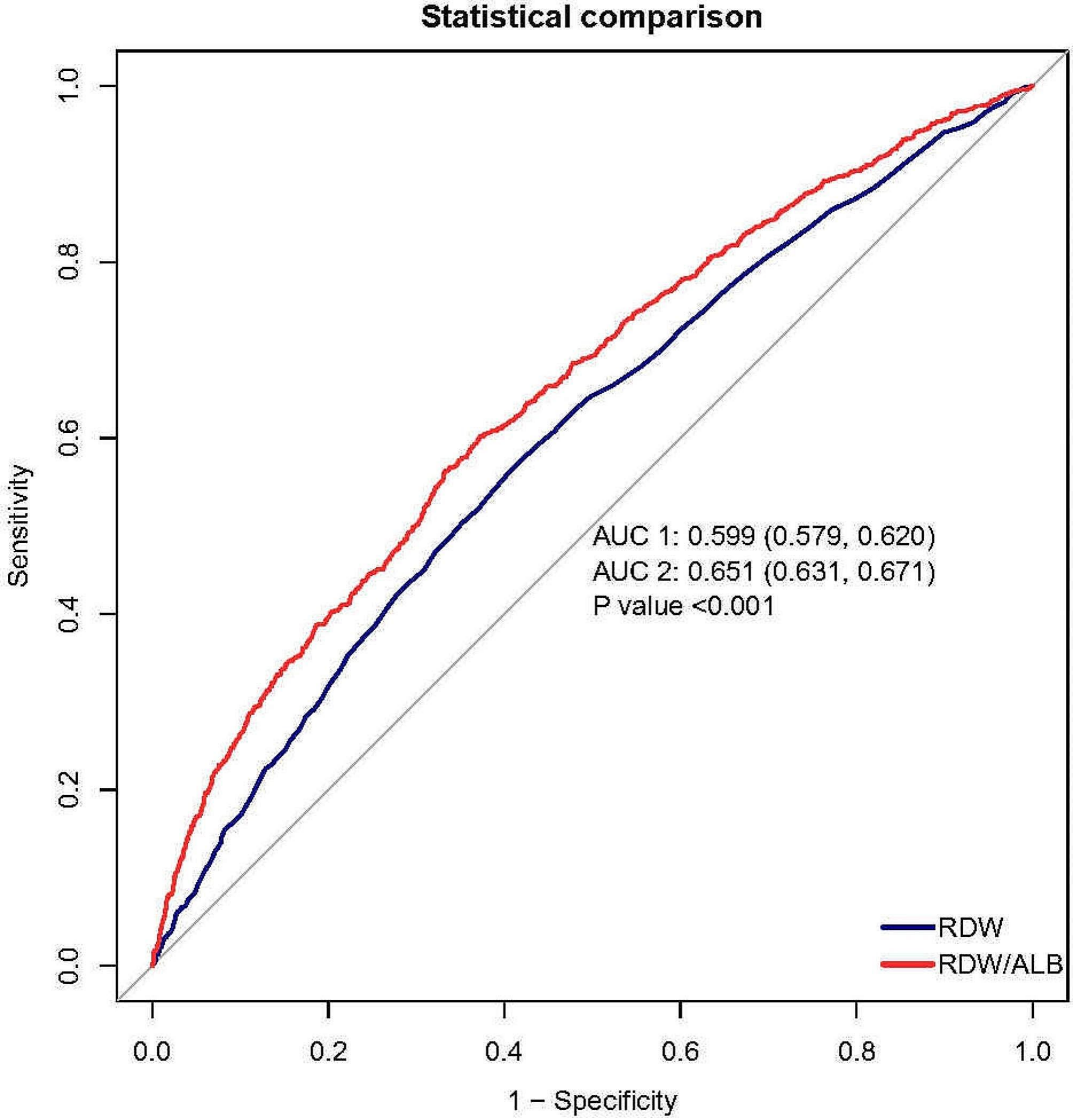
ROC curves of RDW and RAR for in hospital mortality of patients with atrial fibrillation ROC, receiver operating characteristic; RDW, red cell distribution width; RAR, RDW to albumin ratio

The ROC curve comparative analysis demonstrated that the RAR exhibited a superior discriminatory capacity compared to RDW. (Fig. 4). The area under the ROC curves (AUCs) for RAR and RDW were 0.651 (95%CI: 0.631–0.671) and 0.599 (95% CI: 0.579–0.620), respectively (*p* < 0.001).

## Discussion

This study showed a significant positive linear association between RAR and in hospital mortality in patients with AF. With each one-unit increase in RAR, there is a 12% elevation in the in-hospital mortality rate (95% CI 1.06–1.19). Even after adjusting for potential confounding factors, RAR remains independently correlated in hospital mortality among patients with AF. ROC curves suggest that RAR exhibits superior predictive capability for in-hospital mortality in ICU patients with AF compared to RDW. The subgroup analysis did not unveil any interaction within subgroups.

Previous studies have indicated the high prevalence of AF in critical care patients, establishing it as a prognostic marker associated with increased mortality [24, 25]. Inflammation plays an important role in the development and progression of atrial fibrillation [26]. Traditionally, inflammation has been attributed to cytokines production by infiltrating white cells in response to tissue injury and/or immune cell reactions. However, emerging evidence suggests that other cell types, including cardiomyocytes, fibroblasts, and adipocytes, may contribute to the inflammatory signaling pathways associated with atrial fibrillation. Several inflammatory markers, such as C-reactive protein (CRP), tumor necrosis factor (TNF)-α, interleukin (IL)-2, IL-6, and IL-8, have demonstrated associate with the presence or outcome of AF and can impact AF through mechanisms like endothelial damage and platelet activation [26–28]. Although the precise mechanisms through which inflammation affects the clinical presentation and outcomes of AF patients remain incompletely understood, it is recognized that inflammation contributes to atrial remodeling, —structural and functional changes associated with AF development, raising the risk of adverse outcomes, including stroke and mortality.

Red Cell Distribution Width (RDW) is a hematological parameter that is often elevated in the setting of inflammation and oxidative stress. RDW is currently associated with several diseases, including cardiovascular diseases such as stable angina [29], acute coronary syndrome [30], coronary bypass surgery [31], heart failure [32], and stroke [33]. Furthermore, albumin, with its anti-inflammatory, antioxidant, and anti-thrombotic properties, plays a crucial role in cardiovascular health [34]. While RDW and albumin have been shown to be associated with an increased risk of atrial fibrillation [16, 35], RAR, serving as a composite marker, exhibits a superior predictive effect. In recent years, it has been discovered that RAR is associated with type 2 diabetes and foot ulcers [36], chronic kidney disease [20], chronic obstructive pulmonary disease [21], acute myocardial infarction [22] and heart failure [37]. In AF, where inflammatory processes are known contributors, an elevated RDW/ALB ratio may signify a pro-inflammatory state. This could be indicative of a more extensive systemic impact of AF, potentially involving endothelial dysfunction, oxidative stress, and inflammatory pathways. The mechanistic underpinnings of how RDW and serum albumin levels influence in-hospital mortality in AF patients warrant further exploration. Gaining insight into these potential mechanisms has the potential to pave the way for targeted interventions focused on modulating the inflammatory environment and enhancing outcomes in patients with atrial fibrillation.

Our study has certain advantages. Firstly, it stands out as the largest retrospective cohort study examining the connection between the RAR and in-hospital mortality in patients with atrial fibrillation. Additionally, our comparative analysis of the predictive capabilities of RDW and RAR for mortality revealed that RAR outperforms RDW in prognostic accuracy for atrial fibrillation. This finding contributes valuable insights to the clinical diagnosis and prognostication of patients with atrial fibrillation.

The present study has several limitations. Firstly, its exclusive focus on AF patients, limiting generalizability to other populations. Secondly, our findings, derived solely from a single-center evaluation using the MIMIC-IV database, may face limitations in generalizability, the retrospective nature of the study introduces the potential for selection bias. Future research should involve multiple centers and a larger datasets. Thirdly, the measurement of the RAR was performed only once, neglecting the potential impact of varied processes and dynamic changes in AF over time. Future studies should explore the fluctuations in these markers to provide a more comprehensive understanding. Fourthly, we attempted to incorporate a variety of diseases into our exclusion criteria; however, certain conditions such as hematological diseases, infectious conditions, malignancies, were not excluded. And the study may not have considered confounding factors, such as smoking status and atrial volume. Despite these limitations, the prognostic efficacy of RAR for AF patients remains evident.

## Conclusions

This study reveals an independent association between RAR and in-hospital mortality in patients with atrial fibrillation in the MIMIC-IV database. Furthermore, in comparison to RDW, RAR demonstrates superior predictive capabilities. As an inflammatory biomarker, RAR aids clinicians in early and effective prognosis assessment for patients with atrial fibrillation, facilitating prompt intervention and treatment. In conclusion, recognizing the potential role of RDW/ALB in assessing patient conditions can enhance risk stratification, monitoring, and management of individuals with atrial fibrillation.

### Electronic supplementary material

Below is the link to the electronic supplementary material.

Supplementary Material 1

## Data Availability

All data in the article can be obtained from MIMIC-IV database (https://mimic.physionet.org/). To facilitate the reproduction of our results, we provide the list of anonymous patient identifiers for databases in Supplementary data 1.

## Declarations

### Data sharing Statement

The corresponding author will provide the datasets used and analyzed during the current work upon reasonable request.
