# Supplementary material for: Association of red cell distribution width/albumin ratio and in hospital mortality in patients with atrial fibrillation base on medical information mart for intensive care IV database
Source: BMC Cardiovasc Disord. 2024 Mar 21;24:174. doi: 10.1186/s12872-024-03839-6 (PMC10956318; doi:10.1186/s12872-024-03839-6)
Supplement: Supplementary file 1 — Supplementary Material 1 [file 12872_2024_3839_MOESM1_ESM.docx]

Table S1: ICD codes of atrial fibrillation.

| ICD codes | Description |
| --- | --- |
| 42731 | Atrial fibrillation |
| I480 | Paroxysmal atrial fibrillation |
| I481 | Persistent atrial fibrillation |
| I4811 | Longstanding persistent atrial fibrillation |
| I4819 | Other persistent atrial fibrillation |
| I482 | Chronic atrial fibrillation |
| I4820 | Chronic atrial fibrillation, unspecified |
| I4821 | Permanent atrial fibrillation |
| I4891 | Unspecified atrial fibrillation |

Abbreviation: ICD: international classification of diseases.
